# Development of Deep-Learning-Based Single-Molecule Localization Image Analysis

**DOI:** 10.3390/ijms23136896

**Published:** 2022-06-21

**Authors:** Yoonsuk Hyun, Doory Kim

**Affiliations:** 1 Department of Mathematics, Inha University, Incheon 22212, Korea; yshyun21@inha.ac.kr; 2 Department of Chemistry, Hanyang University, Seoul 04763, Korea; 3 Research Institute for Convergence of Basic Science, Hanyang University, Seoul 04763, Korea; 4 Institute of Nano Science and Technology, Hanyang University, Seoul 04763, Korea; 5 Research Institute for Natural Sciences, Hanyang University, Seoul 04763, Korea

**Keywords:** single-molecule localization microscopy, super-resolution microscopy, deep learning, computer vision

## Abstract

Recent developments in super-resolution fluorescence microscopic techniques (SRM) have allowed for nanoscale imaging that greatly facilitates our understanding of nanostructures. However, the performance of single-molecule localization microscopy (SMLM) is significantly restricted by the image analysis method, as the final super-resolution image is reconstructed from identified localizations through computational analysis. With recent advancements in deep learning, many researchers have employed deep learning-based algorithms to analyze SMLM image data. This review discusses recent developments in deep-learning-based SMLM image analysis, including the limitations of existing fitting algorithms and how the quality of SMLM images can be improved through deep learning. Finally, we address possible future applications of deep learning methods for SMLM imaging.

## 1. Introduction

Over the past decade, increasing attention on deep learning algorithms has yielded drastic improvements in multiple fields of research, including image processing. In computer vision, deep learning algorithms have produced significant improvements in image classification, object detection, and segmentation [1]. These advancements have been applied to an unexpectedly wide range of everyday purposes such as facial recognition, autonomous driving, medical imaging, drug synthesis, smart farming, healthcare, retail, inventory management, and advertising [2]. The benefits of deep learning-based methods in a wide range of industrial fields have raised expectations for the fourth industrial revolution. In addition to industrial applications, deep-learning-based methods have attracted considerable attention for applications in many fundamental research fields such as optimization, signal processing, mechanical engineering, medical research, biology, chemistry, and mathematics. In particular, microscopy is an important application of the deep-learning approach. 

Spatial resolution, defined as the shortest distance between two points on a specimen, is the most important feature of optical microscopy [3]. It has long been considered to be physically limited by the diffraction property of light, also known as Abbe’s diffraction limit [4]. An infinitesimal point light source from the sample is transmitted to the image plane through an objective, generating a diffraction pattern of light called the point spread function (PSF) [5]. Since the resolving power of optical microscopy is determined by the minimum distance at which the two PSFs can be resolved, the spatial resolution of optical microscopy is limited by unavoidable blurring due to diffraction limits [6]. To overcome this limitation, various super-resolution fluorescence microscopic techniques (SRM) have recently been developed to allow nanoscale imaging. These techniques can be categorized into two groups: single-molecule localization microscopy (SMLM) and illumination pattern engineering. SMLM employs the photoswitching property of fluorophores to determine each molecule’s location with high precision [7,8]. SMLM methods include stochastic optical reconstruction microscopy (STORM) [9], photo-activated localization microscopy (PALM) [10], and fluorescence photoactivation localization microscopy (FPALM) [11]. In contrast, illumination pattern engineering uses a nonlinear response to fluorophore excitation to overcome the diffraction limit by decreasing the PSF size or modifying the illumination pattern [12]. Such techniques include stimulated emission depletion (STED) microscopy [13] and reversible saturable optical fluorescence transition (RESOLFT) microscopy [14] as negative patterning methods and [saturated] structured-illumination microscopy ([S]SIM) as a positive patterning method [15]. It has been recently demonstrated that SMLM can take advantage of deep-learning-based computer vision algorithms because SMLM techniques rely significantly on computational methods to localize individual fluorophores with high precision through a large number of image frames [16,17,18,19].

Therefore, we focus our discussion on the application of deep-learning methods to SMLM techniques. First, we briefly describe the technical principles and analytical challenges of SMLM, followed by the fundamental components and background of the deep learning approach, as well as traditional and modern computer vision algorithms. Finally, we discuss recent applications of deep learning-based analysis to SMLM and address possible future enhancements. 

## 2. SMLM

### 2.1. Development of SMLM

With the improved resolution of SMLM up to the nanoscale, the technique has been applied to multiple biological processes to obtain molecular ultrastructural information that is inaccessible under diffraction-limited light microscopy techniques. For example, Chan et al. reported purinosome transit in the context of subcellular localization using STORM imaging, which has not been resolved using conventional microscopy [6]. The mechanisms of organelle redistribution and sorting in platelet intermediate maturation and activation processes have also been investigated with the help of nanoscale imaging by STORM [20,21].

Although SMLM has already increased spatial resolution by more than an order of magnitude from the diffraction-limited resolution in conventional light microscopy, numerous attempts have been made to further improve the resolution or obtain multidimensional information from the sample. For example, the dual-objective scheme has been employed for SMLM imaging to obtain a two-fold or higher spatial resolution than previously achieved with single-objective SMLM imaging [22]. This method has also been extended to 3D imaging. Most 3D SMLM imaging experiments have been performed using PSF engineering. For example, 3D STORM imaging was first achieved by employing astigmatism with an axial resolution of approximately 40 nm. By inserting a cylindrical lens in the imaging path, PSFs revealed different ellipticities depending on their axial positions, allowing the determination of axial position with a resolution of ~40 nm [23]. Another implementation for 3D imaging has engineered a double-helical PSF, encoding the z-position in the angular orientation of two lobes in the wide-field image [24]. In addition to 3D imaging, multicolor SMLM for the simultaneous imaging of multiple targets with distinct colors has also been demonstrated using filters, a prism, or a grating. The first demonstration of multicolor STORM imaging employed the combinatorial pairing of activator and reporter dyes and filter-based spectral separation [25]. However, it remains challenging to differentiate more than four colors with a filter due to the overlapping of fluorescence emission spectra of multiple fluorophores. This limitation has recently been overcome with the use of a prism or grating to enable spectrally resolved single-molecule imaging [26,27]. This approach has facilitated crosstalk-free multicolor imaging for four dyes, 10 nm apart from each other in their fluorescence emission spectra [26]. 

Moreover, SMLM has recently been combined with other microscopic and spectroscopic techniques to obtain more information about samples that have not been obtained through SMLM only. For example, SMLM has recently been combined with electron microscopy (EM) to simultaneously obtain the spatial information of specific molecules and the overall cellular ultrastructure [8,28,29]. Furthermore, the integration of SMLM with spectroscopy yields rich spectral information from the single-molecule fluorescence emissions detected by SMLM [7,26]. Due to the multidimensional and functional information obtainable from single-molecule fluorescence emissions, single-molecule spectroscopic imaging has become applicable to multiple areas of chemistry and biology, including single-molecule polarity sensing and molecular dynamics studies [7,27,30]. Thus, recent advancements in the research field of SMLM have made it possible to not only image ultrastructures with near-molecular-scale resolution, but also obtain multi-dimensional information of single molecules that was hitherto unattainable, revolutionizing our understanding of the nanoscale world [7]. 

Collectively, SMLM research has gradually improved over the past several years, greatly facilitating our understanding of various cellular nanostructures that has previously been hindered by the low resolution of diffraction-limited light microscopy.

### 2.2. Principle and Analysis of SMLM Techniques

**Figure 1:**
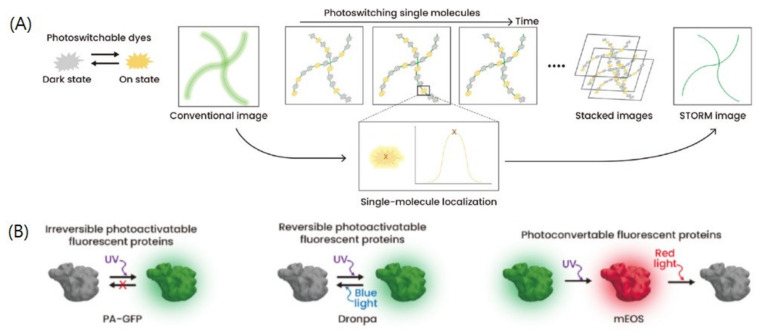
Single-molecule localization microscopy (SMLM). (**A**,**B**) The principles of (**A**) STORM and (**B**) PALM. Adapted from the article of [8] under Creative Commons Attribution (CC BY) license.

In SMLM imaging, fluorophores can be imaged individually through their stochastic on-and-off photoswitching processes; hence, their positions can be determined with high precision in terms of their center coordinates [4,5,31]. The main idea behind SMLM imaging is to avoid the overlapping of PSFs from multiple nearby fluorophores inside the diffraction-limited region. To differentiate between individual molecules, fast photoswitching of fluorophores should be ensured so that only a PSF from a single molecule is imaged at a time [9] (Figure 1). It has been established that fast photoswitching of organic dyes can be achieved through high laser power illumination and a thiol reagent in the imaging buffer [28].

Using their PSFs, the centroids of individual molecules were determined with high precision and reconstructed for a super-resolution image. To determine the centroid positions, each fluorescence image in the raw STORM movie was analyzed for localization. In a fluorescence image, each region where a single fluorophore was switched on was fitted with the following two-dimensional ellipsoidal Gaussian function using nonlinear least-squares regression. The peak from the fitted Gaussian function was considered as the position of each localization [9].

(1) $$I\left(x,y\right)=A+I_{0}e^{\left[-\left(x^{\prime }/a\right)-{\left(y^{\prime }/b\right)}^{2}\right]/2}$$

where,

(2) $$x^{\prime }=\left(x-x_{0}\right)cos\;\theta -\left(y-y_{0}\right)sin\;\theta \;y^{\prime }=\left(x-x_{0}\right)sin\;\theta +\left(y-y_{0}\right)cos\;\theta$$

In this Gaussian function, *x*_0_ and *y*_0_ are the center positions of the peak for the *x*- and *y*-axes, *θ* represents the tilt angle of the ellipse relative to the pixel boundaries, and *I*_0_ is the peak amplitude. *A* is the background level that must be appropriately determined to avoid false single-molecule identification and accurately localize the centroid positions. For example, Chung et al. recently reported that it is important to determine the appropriate background level, particularly for imaging fluorophores with low photon flux, as it is difficult to determine the threshold for effective fluorescence emission above the background level [28]. Localization precision can be approximated by $\Delta =\sqrt{N}$, where ∆ is the width of the diffraction-limited PSF and N is the detected photon number, implying that the precision is mainly determined by the brightness of a fluorophore [5]. The detected photon number also determines the spectral resolution. It has been reported that brighter molecules exhibit similar spectra with an extremely small standard deviation in their spectral mean values, whereas dimmer molecules exhibit a larger variation in spectral mean values owing to their lower signal-to-noise ratios [26]. From the fitting, various data about each PSF of a single-molecule can be obtained, such as the localization coordinates, background intensity, PSF width, number of photons per switching cycle, and switch-on time. 

After identifying single-molecule localizations in each frame, the collected centroid positions were drift-corrected, and the final STORM images were reconstructed. Therefore, it is important to collect a sufficiently large number of high-density localizations to define ultrastructures at a high spatial resolution [29]. The following Nyquist criterion equates image resolution to twice the average distance between neighboring localizations [30].

(3) $$R=2/{\left(\mathrm{localization}\;\mathrm{density}\right)}^{1/D},$$

where R is the image resolution and *D* represents the number of dimensions [29]. This implies that a higher localization density yields higher spatial resolution. However, the collection of more imaging frames can be limited by the quenching process, further deteriorating the time resolution. Consequently, fast switching of fluorophores is required to achieve high spatial and temporal resolution, particularly for live-cell SMLM imaging.

### 2.3. Limitations of SMLM Analysis

As discussed above, the spatial and temporal resolutions of SMLM significantly depend on the image analysis method because the final super-resolution image is reconstructed from the localizations identified through computational analysis. Furthermore, molecular data of individual fluorophores are hidden in the spectral data from PSFs, necessitating the development of a new method for single-molecule spectral analysis of SMLM images. With this aim, we can take advantage of deep learning algorithms to analyze SMLM image data. Existing limitations to current SMLM image analysis methods are expected to be overcome by deep learning. 

First, image reconstruction analysis is expected to be accelerated with the help of deep learning-based analysis [31,32,33]. As described earlier, the final super-resolution image in SMLM is the reconstructed collection of a number of localizations. The reconstruction of super-resolution images with high-density labeling requires a large number of localizations, approximately on the order of millions for microtubule imaging. The issue arises from the fact that each localization is obtained by fitting each PSF with a Gaussian function, which generally requires tens of minutes. In order to analyze super-resolution image data in high throughput, particularly for scanning a wide area of samples, the analysis needs to be accelerated.

Second, the requirement of a large number of localizations limits the temporal resolution of SMLM imaging because sufficient time is needed to acquire abundant localization with high density to successfully reconstruct super-resolution images [34]. Although the temporal resolution of SMLM imaging can be increased by using a high-power laser to accelerate the switching rates of fluorophores and obtain more localizations in a relatively short time, high-power lasers have been reported to cause sample destruction, photodamage, and photobleaching [28,34]. In contrast, the computational strategy is expected to avoid the temporal resolution limits without causing sample damage. For example, a deep-learning-based computational algorithm can reduce acquisition time by reconstructing a high-density super-resolution image from a low-density image [35,36].

Third, localization precision may be significantly improved using deep learning-based analysis methods. As discussed earlier, localization precision is mainly determined by the photon number of the fluorophore. Therefore, various efforts have focused on developing bright fluorophores to increase the spatial resolution of SMLM imaging. However, this approach is limited by the intrinsic photochemical properties of organic dyes. Since a PSF with a high-intensity peak is required to precisely fit a single-molecule localization, this limitation could be avoided with a new deep-learning-based localization method in which precision is not limited by the PSF’s intensity [37].

Fourth, it is challenging to localize densely-spaced overlapping emitters using standard SMLM image analysis. There are several experimental methods to obtain a low density of emitting fluorophores in each imaging frame without overlaps, such as through the use of high laser power or a high concentration of thiol reagents in the imaging buffer [28]. However, these methods may be time-intensive and inefficient in avoiding the overlapping of emitting fluorophores. Therefore, it is necessary to develop a new algorithm to improve localization precision. Although the fitting process of overlapping PSFs in conventional image analysis may require a time-consuming iterative procedure, a deep-learning-based method is expected to overcome this limit and reduce computational cost [19,31].

Lastly, it has been recently suggested that there is still hidden molecular spectral information in PSFs obtained from an unmodified microscope, which cannot be extracted by conventional SMLM imaging analysis [38]. For example, additional optics such as cylindrical lenses, filters, prisms, and gratings have been required to obtain axial localization or color information of single molecules. However, this information may be determined using deep learning from regular SMLM data, which can be obtained without the use of additional complicated optics [38,39,40,41,42]. Such an approach is expected to provide more detail and information regarding individual molecules without requiring a complicated setup.

This review discusses recent technological advances in the application of deep learning to SMLM image analysis by categorizing the challenges of current SMLM analysis. To understand the details of the suggested methods, we first introduce key concepts, architecture, and algorithms in the field of deep learning-based computer vision.

## 3. Deep Learning in Computer Vision

### 3.1. Machine Learning and Neural Network

Machine learning is an application of artificial intelligence that enables systems to learn and improve from experience without being programmed explicitly. This approach focuses on developing an algorithm that can access data and use it to learn. The algorithm can be determined based on the input data, output data, and model. In machine learning, an appropriate function is optimized to predict the desired output from given input data. This function is determined by its shape and coefficients, called the model architecture and weight parameters, respectively. 

A neural network is a machine learning method whose model architecture uses neurons as basic computation units. The general neural network architecture is composed of several layers, in which the individual layer first receives multiple neurons as layer input, followed by designated operations to generate new neurons as layer output. The output can then be used as layer input for subsequent layers. 

The operations in each layer can vary and each layer typically contains weight parameters that are required to compute its output. Therefore, investigating appropriate weight parameters through the training process is the key to finding a well-performing algorithm. 

The fundamental operation of a neural network is a linear operation. In a linear layer, each output neuron is obtained by a linear combination of input neurons with layer weight parameters as their coefficients and an additional single-bias term. To impose non-linearity, an activation function such as Rectified linear unit (ReLU) [43] or sigmoid is followed by a computation. A linear layer is also called a fully-connected layer because each output neuron relates to all input neurons. 

The convolution operation is used more commonly than the linear operation for processing image and video data. The inputs and outputs of the convolution operation are three-dimensional vectors with width, height, and depth (channel). Each convolution has a small-sized filter composed of weight parameters that act on the input data in a sliding window fashion to generate an output neuron. This preserves the width and height structure of the input data; thus, important features can be captured more easily by preserving the image structure in the data forward path.

### 3.2. Notable Model Architectures in Deep Learning

The type of input and output data in a machine learning algorithm generally depends on the algorithm’s purpose. Among the various purposes, classification and regression are considered major problems in prediction. Classification is the process of finding or discovering a model or function that separates the input data into multiple categorical classes. The most common method to handle model output for classification involves finding a probability map for each class and selecting the category with the maximal probability value. Therefore, to classify given data into N-specific classes, a model is designed to produce a vector consisting of N real numbers as the final output. Regression approximates a mapping function from the input data to continuous output data. The number of output values must be equal to the required number of predictions, but the output shape can be an array of any number of dimensions, depending on the algorithm’s purpose. 

**Figure 2:**
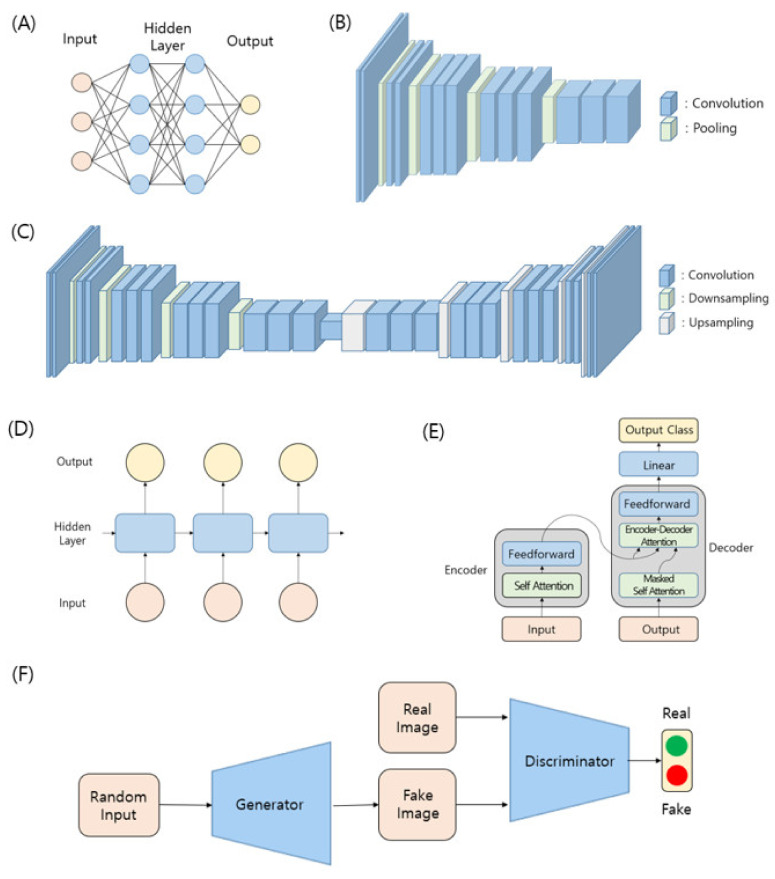
A visualization of deep learning architectures. (**A**) Multi-Layer Perceptron. (**B**) CNN based feature network [44]. (**C**) Encoder–Decoder architecture. (**D**) Recurrent neural network. (**E**) Transformer [45]. (**F**) Generative adversarial network [46].

The choice of model architecture, determined by the type of input and output data, is often crucial for algorithm performance. Two different algorithms with different problem setups and purposes can often employ similar architectures because of the shapes of the required output data being similar. Herein, we introduce several common model architectures used in image processing with deep neural networks (Figure 2). The study of these architectures is the key to understanding the applications of deep learning-based algorithms in super-resolution microscopic imaging, which is our focus in this review.

#### 3.2.1. Multilayer Perceptron (MLP)

MLP is the oldest model architecture in deep learning. As an elementary architecture, MLP consists of several linear layers. The number of input neurons is the size of the input data, and the number of output neurons is the size of the desired output. The number of intermediate or hidden layers, and the number of neurons in each hidden layer, determine the entire architecture of MLP. 

MLP has been used in early neural networks, usually with only a small number of hidden layers. Input data for MLP needs to be flattened to single-dimensional vectors, making it difficult to maintain the structure contained in the data itself, such as location information near pixels in an image or time information in sequential data. Nevertheless, for simple datasets such as MNIST (Modified National Institute of Standards and Technology) [47], linear operations are sufficient to obtain satisfactory classification accuracy. If the input data are not too complex, the use of a small, shallow MLP can help solve classification or regression problems.

#### 3.2.2. CNN-Based Feature Network

To exploit the geometric structure of image data, the use of convolutional operations, rather than linear ones, is desirable for solving computer vision problems. CNNs (convolutional neural networks) incorporate a neural network architecture whose primary operations are convolutions. To achieve high performance in several vision tasks, the neural network architecture first needs to capture common, important features in training images while eliminating unwanted noise information as the neural-network operations proceed. This part of the architecture is called the feature network. Generally, a neural network architecture is composed of feature network parts and task-specific parts. Input image data are fed into the feature network first and then the output is used for the following task-specific component. A single feature network architecture can be exploited for different vision tasks. Therefore, the development of a superior feature network is essential to improve the accuracy of general computer-vision algorithms.

The first work on modern CNNs was conducted in the 1990s. LeCun et al. suggest that a five-layer CNN model that aggregates simpler features into more complicated features, can be successful for handwritten digit recognition [47]. In 2012, CNN-based AlexNet achieved state-of-the-art performance on ImageNet, which is the most famous classification benchmark in the computer vision field [48,49]. Ongoing research on CNNs has been flourishing since then, and several architectures with a bit deeper layer, such as the ZF-Net and VGG (Visual Geometry Group) [44,50]. GoogLeNet introduced the “Inception module” designed to apply parallel convolution operations with various filter sizes on the layer input and concatenate all outputs together, channel-wise [51]. Further investigation and improvement of the inception module has led to the development of enhanced architectures such as Inception V2, V3, and V4 [52,53]. 

ResNet uses residual connections to construct an even deeper architecture [54]. It tries to overcome potential difficulties in optimization for deep neural networks by copying the learned layers form the shallower model and setting additional layers for identity mapping. The residual connection was found to be very effective for stable training and it had a significant impact on further studies. 

Research on CNN feature networks have flourished with various creative approaches. For example, SENet includes a feature recalibration module that adaptively reweighs feature maps [55]. DenseNet uses dense blocks where all layers are interconnected in a feedforward fashion [56]. 

EfficientNet increases network capacity by scaling the width, depth, and resolution, while balancing the accuracy and efficiency [57]. It searches for the optimal set of compound scaling factors given a computation budget and scales up the architecture to satisfy the given condition by using smart heuristic rules. EfficientNet achieved state-of-the-art performance on many classification benchmarks.

#### 3.2.3. Encoder–Decoder Architecture

Generally, during the operation of a CNN feature network, the width and height of the input data decrease, while its channel (depth) increases. In many applications such as semantic segmentation, it is required for the spatial resolutions of the input and output to be the same. For example, the channel dimension of one output pixel can hold the classification results for the corresponding input pixel at the same spatial position. The common method is to construct an architecture, referred to as the encoder–decoder architecture, that can upsample output of the feature network by enlarging its width and height. Although the terms encoder and decoder are generally used in machine translation, they have roles similar to those in computer vision problems.

The decoder is typically composed of several convolutional layers and an upsampling layer. Although there are several choices for upsampling features, such as the usual bilinear interpolation or unpooling, the modern method uses a transposed convolution operation. Transposed convolution, sometimes called deconvolution, can be considered a special case of ordinary convolution where the output feature size is greater (usually doubled) than that of the input feature. This operation also has a learnable parameter that enables the network to optimize feature scaling using data. 

The Fully Convolution Networks (FCN) was one of the earliest works to adapt the encoder–decoder architecture. It uses several transposed convolutions to upsample the intermediate feature map [58]. SegNet and DeconvNet suggest an unpooling method that remembers the position of each maximum activation value when doing max pooling in the encoder part [59,60]. Hourglass adds a residual connection between the encoder and decoder components [61]. U-Net suggests a decoder whose upsampling method is a transposed convolution with its own encoder architecture and residual connection [62].

#### 3.2.4. Recurrent Neural Network (RNN) and Transformer 

RNNs are a class of neural networks that allow previous outputs to be used as inputs with hidden states. For each time step in the vanilla RNN, the network produces the activation and output using the activation from the previous time step. This approach is helpful in modeling sequence data and is primarily used in the fields of natural language processing and speech recognition. 

Vanishing and exploding gradient phenomena are often encountered in standard RNNs. Long Short Term Memory (LSTM) is explicitly designed to avoid long-term dependency problems such as that of vanishing gradients [63]. The gated recurrent unit (GRU) incorporates two gate operating mechanisms, called the update gate and reset gate, to solve the problem of a standard RNN [64].

In 2017, a transformer was introduced to replace RNN models [45]. This transformer adopts the mechanism of self-attention, differentially weighting the importance of each component of the input data. The core architecture comprises a stack of encoders that are fully connected to a stack of decoders. The transformer achieved state-of-the-art performance in natural language processing and has recently been applied to image processing with results competitive with those of convolutional neural networks. Vision Transformer (ViT) represents an input image as a series of image patches, such as the series of word embeddings used in transformers to text, and directly predicts class labels for an image [65]. Further research on vision transformers is flourishing on all tasks in computer vision, including image classification, object detection, and segmentation.

#### 3.2.5. Generative Models and Generative Adversarial Networks (GANs)

Generative modeling is an unsupervised learning task in machine learning that involves the automatic discovering and learning of regularities or patterns in input data. Typically, a generative model describes how a dataset is generated in terms of a probabilistic model. With the rise of deep learning, deep generative models have been formed through a combination of generative models and deep neural networks. An increase in the scale of neural networks is typically accompanied by an increase in the scale of the training data, both of which are required to achieve high performance. 

Although variational autoencoders (VAEs) and pixel RNNs/CNNs are some of the popular deep generative models, deep generative models have exhibited remarkable success since the introduction of GANs [46,66,67,68]. GANs employ a discriminator network with a generator network. The discriminator network is used to distinguish between real and synthetic images, whereas the generator network attempts to fool the discriminator by generating real-looking images. Overall, a generative network learns to map from a latent space to a probability distribution of the data of interest, whereas a discriminative network distinguishes candidates created by the generator from the true data distribution. For image generation, the generator is typically an up sampling network with transposed convolution, whereas the discriminator is a regular convolutional neural network.

### 3.3. Algorithms in Computer Vision

**Figure 3:**
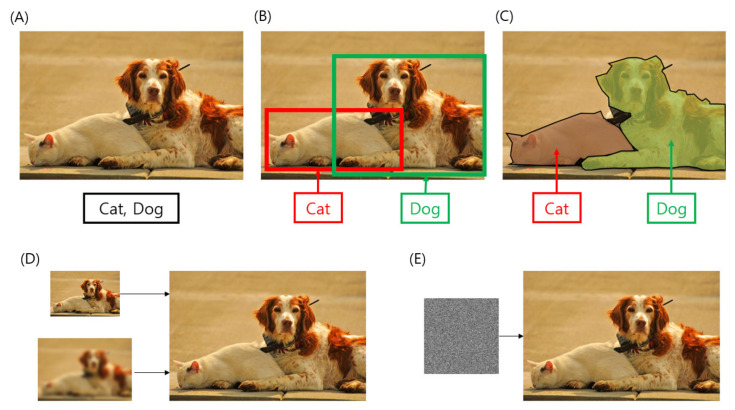
A list of computer vision algorithms. (**A**) Image classification. (**B**) Object Detection. (**C**) Semantic segmentation. (**D**) Image reconstruction. The top represents a super-resolution algorithm, while the bottom represents an image deblurring. (**E**) Image generation from random noise. The sample image is brought from MS COCO dataset provided under a Creative Commons Attribution 4.0 License [69].

The architectural design must be determined carefully depending on the objective of the vision algorithm. For each notable vision algorithm (Figure 3), there are general architectural choices to accomplish this goal. To improve the accuracy or speed of the algorithm, simple modifications, and additional structures may be helpful. 

#### 3.3.1. Classification

The classical problem in computer vision is to determine whether the image data contains a specific object, feature, or activity. The image classification problem involves assigning an input image to one label from a fixed set of categories. This is one of the core problems in computer vision, which, despite its simplicity, has a large variety of practical applications. More complicated computer vision tasks, such as object detection and segmentation, can be reduced to image classification tasks. 

The general architecture for image classification algorithms is composed of feature networks and a small number (typically two or three) of additional fully-connected or convolutional layers. The output of the entire network is a single-dimensional vector, whose magnitude is the same as the number of classes. Given that the performance of image classification algorithms mainly depends on the feature network, the image classification benchmark is often used to compare feature networks. Thus, the developments in feature networks and classification are compatible in this sense. 

The largest image classification benchmark dataset is ImageNet, and previously mentioned feature networks such as AlexNet, VGG, GoogLeNet, ResNet, and SENet achieve top-level performance [50,52,53,56,57]. DenseNet and EfficientNet have also exhibited strong classification performance [56,57].

Recently, transformer-based feature networks such as ViT have been actively studied for vision tasks, and many outperform CNN-based feature networks. The original ViT uses uniform-scale image patches for its self-attention mechanism, making it difficult to aggregate local and global features. The Pyramid transformer (PvT), as well as Swin Transformer V1 and V2, suggest the use of multi-scale image patches to exploit both low- and high-level information, as in CNN feature networks [70,71,72]. 

#### 3.3.2. Object Detection

A regression algorithm that extracts certain continuous values from an image is often required for tasks such as object detection. Object detection is the task of recognizing instances of a predefined set of object classes and describing the locations of each detected object in the image using a bounding box. The output of an object detection algorithm is a list of boxes with a class probability map and four coordinates (typically x and y coordinates of the top-left corner, and the width and height of the box). 

The most well-known CNN-based object detectors are the region convolutional neural network (RCNN) families [73]. RCNN employs external region proposal algorithms, such as selective search [74]. Each proposed region is re-scaled to a specific size and used as input of the CNN feature network. Fast R-CNN was designed under the assumption that CNN feature network operations for each region are redundant and inefficient [75]. In Fast R-CNN, the input image is fed into the CNN feature network first, and the generated convolutional feature maps are used to extract the region of proposals. Faster-RCNN suggested using Region Proposal Network (RPN) to accelerate the algorithm while improving accuracy [76]. Mask-RCNN uses an additional head for binary mask prediction and upgrades RoI pooling to RoI alignment to produce an additional improvement in accuracy [77].

Prior object detection algorithms use regions to localize the object within the image first, and then re-calibrated the object location. In this sense, we refer to these object detection algorithms as two-stage detectors. In contrast, one-stage detectors typically adopt a straightforward fully convolutional architecture and output a classification probability map and box offsets at each spatial position. One-stage detectors are more efficient and faster, whereas two-stage detectors have advantages in terms of accuracy. 

You only look once (YOLO) is an early model of a one-stage detector that uses only convolutional layers [78]. RetinaNet uses focal loss and a feature pyramid network to solve the class imbalance problem and enrich features obtained from the feature network [79]. Fully Convolutional One-Stage Object Detection (FCOS) checks the centeredness of each spatial position to predict box location more rigorously [80]. 

Recently, transformers have been used to propose new architectures for object detection. DETR suggests using both encoder and decoder architectures in transformers by assigning the decoder tokens a role to guess the box location and class [81].

#### 3.3.3. Semantic Segmentation and Image Reconstruction

Image segmentation can be formulated as a classification problem of pixels with semantic labels (semantic segmentation) or the partition of individual objects (instance segmentation). Semantic segmentation requires dense prediction, which necessitates a class map for each pixel for the input image. Therefore, the most common design choice is encoder–decoder architecture in modern deep neural networks. 

The encoder–decoder architectures introduced earlier, such as FCN, DeconvNet, SegNet, and U-Net, all attempt to solve the semantic segmentation problem for specific applications [58,59,60,62]. V-Net is another FCN-based model with a novel objective function based on the Dice coefficient, enabling the model to handle the imbalance between the number of foreground and background voxels [82].

There are many other approaches to achieve optimal performance in segmentation. The pyramid scene parsing network (PSPN) is a multi-scale and pyramid-network-based segmentation model that aggregates the different sub-region representations obtained by the CNN feature network [83]. DeepLab (v1, v2, v3, v3+) utilizes dilated convolution (“astrous” convolution) to address the decreasing resolution in the networks [84,85,86].

The architecture for semantic segmentation can be used for other dense prediction tasks with minor modifications. Key-point detection (pixel-level localization), flow estimation, and depth estimation algorithms are all tasks that require the prediction of real numbers or vectors on each pixel in the input image, like segmentation. Moreover, the image reconstruction tasks also take advantage of the similar architecture. Making a low-resolution image into a realistic high-resolution image (this algorithm is called super-resolution in the computer vision area) or changing a blurred image into a clean image (image deblurring) are such examples.

#### 3.3.4. Image Generation

Image generation has various applications, one of which is to assist the development of other algorithms. For example, image generation can be applied to unsupervised learning by synthesizing training data when the existing training data is limited. GANs have exhibited state-of-the-art performance in most of image generation tasks and among modern image generation architectures consequently based on the GAN architecture.

Research on GANs has enjoyed considerable popularity since 2017, with DCGAN, CoGAN, progressive GAN, CycleGAN, and StyleGAN being several popular architectures [87,88,89,90,91]. Applications of GANs have rapidly increased to include fashion, advertising, science, video games, and audio/image synthesis. 

## 4. Application of Deep Learning to SMLM Image Analysis 

Based on the previously described background of SMLM and deep learning algorithms, we discuss the recent technological advances in the application of deep learning to SMLM image analysis by focusing on the challenges of traditional SMLM analysis, summarized in the first section of this review (Table 1).

### 4.1. Fast Single-Molecule Localization Using Deep Learning 

In SMLM, the locations of individual emitters are generally obtained by fitting each PSF with a Gaussian function, which incurs a high computational cost to handle the vast amounts of data produced by the SMLM microscope. Therefore, the demand for fast algorithms to localize more than ten thousand molecules is increasing. The general fitting method for single-molecule localization requires the optimization of sample-dependent fitting parameters, such as the background level and PSF width, which requires additional time for precise localization. Furthermore, multi-emitter fitting to analyze a highly dense region with overlapping emitters poses an algorithmic challenge that requires complex mathematical computations. To overcome this limitation, various deep learning-based localization methods have recently been developed by several research groups. 

**Figure 4:**
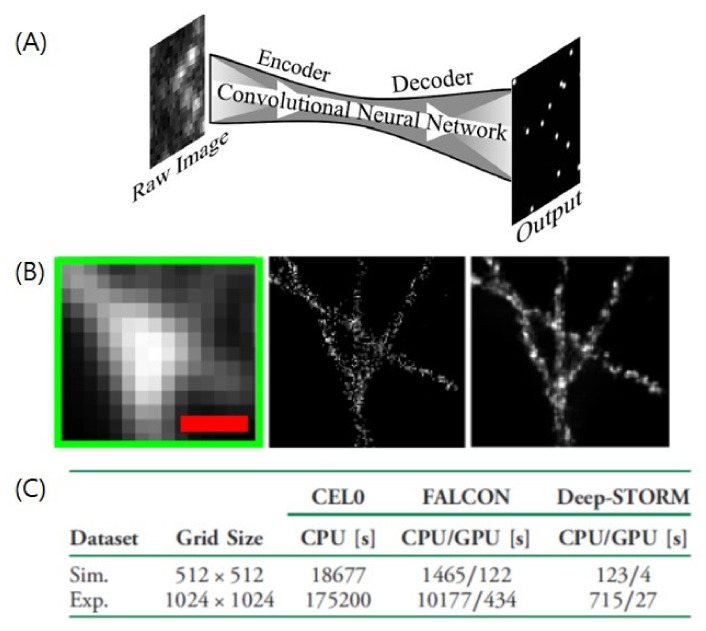
A deep-learning-based method for the fast single-molecule localization (**A**) Network architecture of Deep-STORM. A set of diffraction-limited images of blinking emitters is fed into the convolutional neural network to generate the final super-resolved image. (**B**) Experimental microtubule images. (Left) Diffraction-limited low resolution image. (Middle) Reconstructed image by the CEL0 method. (Right) Reconstructed image by Deep-STORM. Scale bar is 2 μm. (**C**) Comparison of runtime between different methods. Adapted from the article of [31] under the OSA Open Access Publishing Agreement.

The first deep-learning-based approach for SMLM image analysis was Deep-STORM [31] (Figure 4). Deep-STORM is a parameter-free super-resolution image reconstruction method that uses an encoder–decoder architecture with a convolutional neural network. Instead of localizing emitters explicitly, the network creates a super-resolved image directly from the raw data. To train the data, pairs of a diffraction-limited image and the corresponding super-resolved image are first generated by PSF simulations and then tested with both simulated data and experimental microtubule images. Deep-STORM has been demonstrated to be significantly faster and more accurate than conventional algorithms, including accelerated sparse-recovery methods and CEL0 [31].

DeepLOCO is another fast 3D localization method that uses neural networks [32]. DeepLOCO trains a neural network that maps a single frame to a list of localizations. In this method, the network architecture is comprised of a convolutional neural net for feature extraction and an additional fully-connected layer with a residual connection. It generates 2D or 3D coordinates of PSFs directly as outputs, without strong assumptions on the forward and noise models. The end of the neural network consists of two heads: location and confidence. DeepLOCO generates training images by simulating various types of mathematically generated PSFs and adding noise. DeepLOCO has been demonstrated to be substantially faster than existing algorithms while maintaining localization precision. Thus, it was reported that DeepLOCO enables the analysis of a 20,000-frame experimental 3D SMLM dataset within approximately one second [32].

Zelger et al. also used a CNN architecture to directly predict the 3D coordinates of PSFs from a single image for a parameter-free image analysis [33]. Simulated images of emitters at random 3D positions were used to generate the training data. The localization precision in this method was shown to be comparable to that of the existing approach, whereas the speed of the algorithm was much faster than maximum likelihood estimation (MLE) fitting. For example, the algorithm was demonstrated to perform with approximately 22k localizations per second, being more than three orders of magnitude faster than the MLE algorithm of ThunderSTORM; hence, it enabled video-rate image reconstruction for biology dynamics [33]. Furthermore, Zelger et al. experimentally demonstrated the feasibility of jointly estimating Zernike mode magnitudes for aberration modeling and found that the accuracy of estimates can be significantly improved [33].

### 4.2. Constructing High-Density Super-Resolution Image from A Low-Density Image 

Traditional single-molecule localization methods often require a large number of frames and localizations to reconstruct a super-resolution image with high density, which requires an intensive acquisition time. Recent deep-learning-based computational algorithms avoid this issue by enabling the reconstruction of a super-resolution image with a high density from a low-density image, reducing the acquisition time.

For example, Ouyang et al. developed ANNA-PALM using a neural network to generate SR images with a much smaller number of raw frames [35]. The network architecture of ANNA-PALM is based on pix2pix and the generator uses the U-Net architecture [62,92]. For training, ANNA-PALM uses a sparse SR image from a small subset of the frames in the entire dataset and a dense SR image from the entire frame. Widefield and sparse images were used as inputs for the generator to output the prediction image. The predicted output image was compared with a real dense SR image using several different errors, including the conditional GAN error. During the test phase, only the generator part can be used to generate highly dense SR images with a small number of frames. By demonstrating simulated and experimental images of various cellular structures such as microtubules, nuclear pores, and mitochondria, the authors showed that super-resolution images could be obtained from up to two orders of magnitude fewer frames than with traditional methods while achieving a comparable spatial resolution [35]. They also demonstrated that the super-resolution image could be reconstructed from widefield images alone, enabling high-throughput live-cell super-resolution imaging with high speed [35].

**Figure 5:**
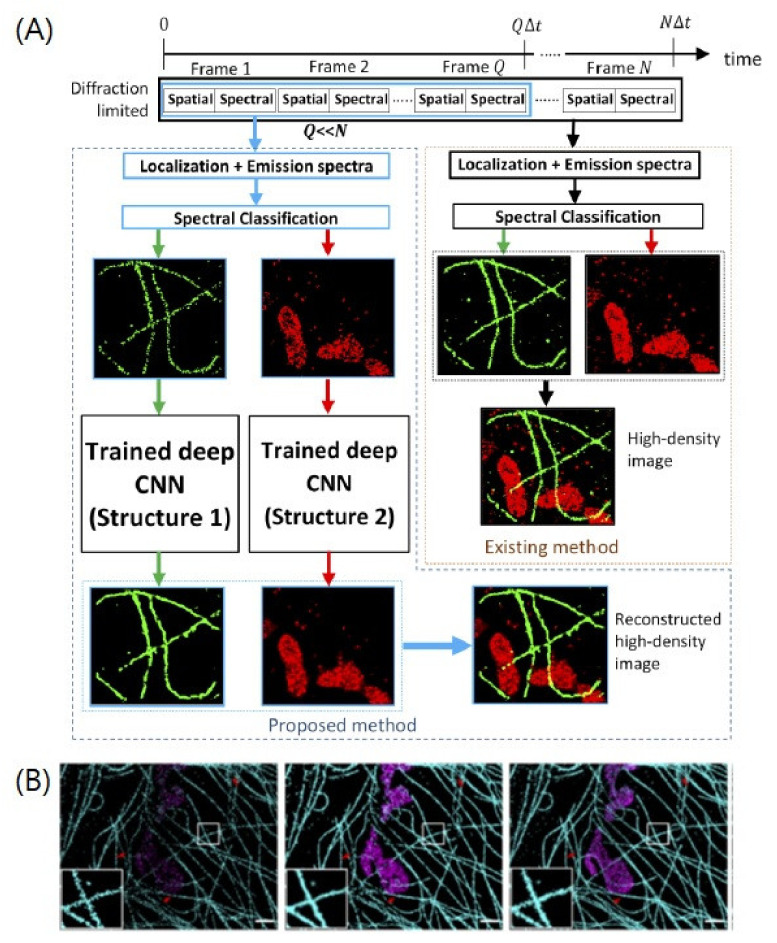
A deep-learning-based method for constructing high-density super-resolution image from a low-density image. (**A**) The trained deep CNNs reconstruct the high-density multicolor image using a small number of image frames. (**B**) The two-color STORM images of AF647 labeled tubulin (cyan) and CF660C labeled mitochondria (magenta) in a COS-7 cell. (Left) Reconstructed image from 3000 frames by existing fitting method. (Middle) Reconstructed image from 3000 frames by deep CNN. (Right) Reconstructed image from 3000 frames by existing fitting method from 19,997 frames. Scale bar = 1.5 μm. Adapted from the article of [36] under the OSA Open Access Publishing Agreement.

Recently, GAIRE et al. introduced another CNN-based method to generate a high-density SR map with a rather simple reconstruction architecture compared to ANNA-PALM [36] (Figure 5). The small frames of the entire dataset were used to find individual PSFs and spectra, and each individual PSF map was fed into a spectrum-specific reconstruction network. The reconstruction architecture was composed of several convolution operations with residual connections without downsampling or upsampling. The training data were generated from the experimental data using the established SMLM algorithms. Random frame sampling, data augmentation, and random cropping were applied to increase the number of training inputs. With this approach, the author demonstrated that super-resolution images could be reconstructed with high quality by using up to 8-fold fewer frames than typically needed. Consequently, multicolor SMLM imaging has been successfully performed for tubulin/mitochondria, peroxisome/mitochondria, and tubulin/mitochondria/peroxisome, with a significantly reduced acquisition time.

### 4.3. Improvement of Localization Precision 

In SMLM imaging, the localization precision of a single molecule is the most important factor to determine the microscope’s spatial resolution. Since localization is identified by fitting algorithms, its precision from given single-molecule image data is expected to be further improved by deep-learning-based computational algorithms. 

**Figure 6:**
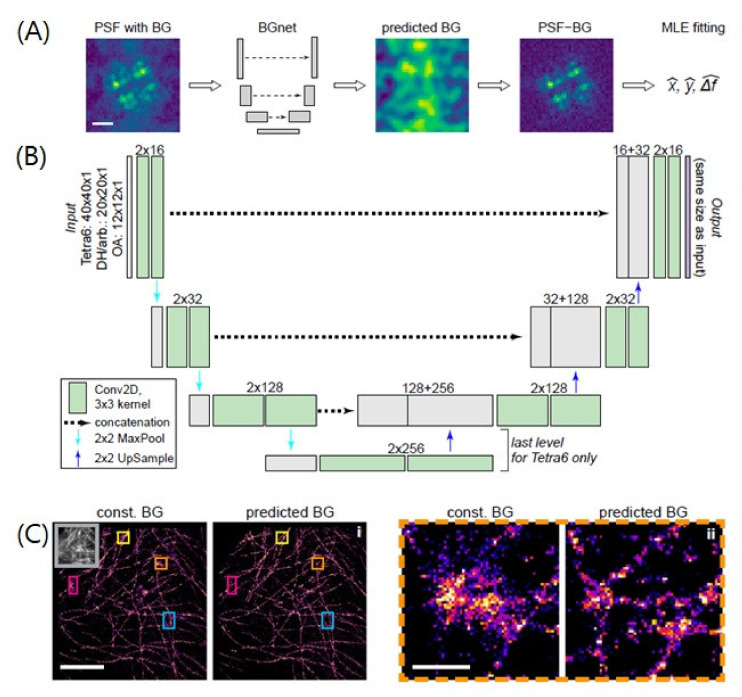
A deep-learning-based method for improving the localization precision. (**A**) The entire process of BGnet. The predicted BG can be readily subtracted from the input PSF image by BGnet and it is used to generate the BG-corrected PSF for subsequent analysis, for example via MLE fitting for position estimation. Scale bar: 1 μm. (**B**) Network architecture of BGnet. (**C**) (Left) Super-resolution images of microtubules in fixed BSC-1 cells using the BG correction with a constant BG estimate or with BGnet. (Right) The magnified images of the boxed region are shown on the right. Scale bars: 5 μm for the left images and 500 nm for the right images. Adapted from the article of [37] under Creative Commons Attribution (CC BY) license.

For example, the recently developed deep neural network, BGnet, yielded substantial improvements in localization precision compared to existing fitting methods (Figure 6). 

To accurately identify the centroid position of the PSF with high precision, it is important to first obtain background-corrected PSF images. Thus, it is critical to accurately estimate the background to extract the maximum information from single-molecule images [37]. However, it is often not straightforward to determine the appropriate background fluorescence for each PSF image, as the local background signal varies [28]. To overcome this limitation, Mock et al. employed a U-net type neural network called BGNet with the aim of reducing the correct background of PSF images for high localization precision. The network received corrupted PSF images and was trained to directly output the background image with intensity. By removing the predicted background from the original images, clearer PSF images could be used for the established MLE-fitting super-resolution algorithms. The training data were generated by PSF simulations with various PSFs and Perlin noise. Thus, BGnet successfully demonstrated an improvement in the localization accuracy of single molecules with various background complexities for both simulated and experimental data. For instance, they showed that the structured background estimated with BGnet resulted in a higher quality of super-resolution images of microtubules, which was not resolved by a constant background estimate. 

### 4.4. Localization of Overlapping PSFs

The main principle of the SMLM is to separate each PSF by employing the photoswitching properties of the fluorophores. Although these properties are experimentally controllable to some extent, their stochasticity often makes overlapping unavoidable, particularly for high-density regions. The localization of such overlapping PSFs is highly challenging with traditional fitting algorithms, as it requires a time-consuming iterative procedure. In contrast, recently developed deep learning-based methods have shown promising results by generating high-quality super-resolution images at ultra-high labeling densities.

Deep-STORM has successfully achieved the reconstruction of super-resolution images for regions with a high density of overlapping emitters using a deep convolutional neural network with high precision [31]. By creating a super-resolution image from the raw data directly instead of explicit localization of emitters, a high-density region can be reconstructed much faster than with prior fitting algorithms. For example, Deep-STORM was demonstrated to perform well up to a density of ~6 [emitter/μm^2^], which is comparable to the highest performance of conventional multi-emitter fitting methods [31]. 

**Figure 7:**
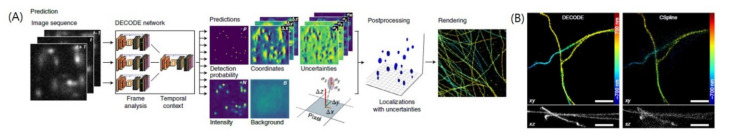
A deep-learning-based method for localization of overlapping PSFs. (**A**) The network architecture of DECODE. Using the information from multiple image frames, DECODE predicts output maps representing the detection probability, subpixel spatial coordinates, brightness, uncertainty, and an optional background for each pixel. (**B**) Comparison between the performances of the DECODE and the CSpline algorithm for the high density, low signal double-helix challenge training data. Scale bars, 1 μm. Adapted with permission [19].

Recently, Decode (deep context-dependent) has also been developed as a deep-learning-based method for high-density PSF images [19] (Figure 7). Decoding differs from traditional localization algorithms by simultaneously performing detection and localization of emitters. It also has the flexibility to determine a wide range of imaging parameters such as 3D positions. The network architecture is composed of two stacked U-nets. The first stage computes the feature representation of a single frame, and the second stage uses integrated feature representations of the consecutive frames to produce the final predictions. The final output comprises multiple channels of the same size as the input image, indicating the probability of being an emitter, brightness, 3D coordinates, background intensity of each pixel, and uncertainties for localization and brightness. This extra pixel-level information aids in rendering the super-resolution image. For training data, Decode uses simulated PSFs with the loss function obtained by comparing the true and predicted data for counts, locations of emitters, and background images. This computational tool for localizing single emitters at high density with high precision also successfully demonstrated the fast dynamics of microtubules in live cells with reduced light exposure and ultra-high labeling density [19]. Thus, this method is expected to not only save imaging times but also increase localization density in SMLM. 

### 4.5. Extracting Additional Spectral Information from PSF 

PSFs typically conceal rich and multidimensional information such as identity, lateral and axial location, and emission wavelength. Several research groups have employed deep neural networks to extract this hidden information from the diffraction-limited image of a fluorophore. 

Zhang et al. developed a deep neural network for multiplexed single-molecule analysis, referred to as smNet [39]. This network accepts individual PSFs as input to extract a 3D location, orientation, and wavefront distortion. It uses a ResNet-like architecture with 27–36 layers and achieves high performance in terms of precision, accuracy, and speed. The training data were obtained by PSF simulation with a wide range of positions, signals, and background levels for more realistic data. By demonstrating smNet using both simulated and experimental datasets, the authors showed that highly multiplexed physical and physiological information could be extracted from the emission pattern of a single molecule.

More recently, Nehme et al. developed a new approach called DeepSTORM3D that localizes multiple emitters with densely overlapping PSFs over a large axial range by encoding the emitters’ axial positions from multiple 3D PSFs [42]. They also employed convolutional neural network architecture to estimate the 3D coordinates of PSFs from a camera frame with multiple 3D PSFs [42]. The architecture was composed of two components. The simulated 3D emitter positions were fed to the first component to simulate a low-resolution camera image. This image was then fed to the second component that attempted to recover the simulated emitter positions. The first component obtains a more optimal PSF shape, enabling the precise estimation of 3D localization in the second component. Using this network to design the optimal PSF for the multi-emitter case, Nehme et al. demonstrated super-resolution reconstruction of mitochondria and volumetric imaging of telomeres in cells.

Kim et al. used two neural network architectures to predict the axial position and emission color of a single molecule from its PSF [38]. Each neural network was composed of fully-connected layers: one for color classification, and another for axial position estimation. The weight parameter for the second network was trained and determined separately for the color types. This method was demonstrated for fixed cells by extracting the color and axial position information of single molecules from regular localization microscopy data. This method simplifies the experimental implementation without the need to modify the PSF shape or divide the single-molecule fluorescence emission into different optical paths.

Similar to the work by Kim et al., Hershko et al. used a CNN architecture to classify the color channels of individual PSFs [40]. Furthermore, they attempted to determine the optimal PSF shape that achieved both accurate localization and color classification of emitters. The proposed method was comprised of two components: the SLM optimizer that produced PSFs from simulated point sources of different colors, and the reconstruction network that generated 2D coordinates of PSFs with the color channel by using an encoder–decoder architecture with convolution and transposed convolution operations. These two architectures were jointly trained using simulated training data. By exploiting the chromatic dependence of the PSF, the neural network was demonstrated to determine the color of an emitter from a grayscale camera image obtained by a standard fluorescence microscope without PSF engineering. They also developed an additional neural net to generate a color-encoding PSF using phase modulation to further improve the color differentiation between multicolor species.

Deep learning has also been employed in the recently developed spectrally-resolved SMLM method (SR-SMLM) [41]. In SR-SMLM, single-molecule spectra can be obtained with localizations by inserting a prism or transmission grating [26,27]. Although this new single-molecule spectroscopic technique provides multidimensional and functional information about individual molecules, which have been generally masked in the ensemble averages of conventional bulk spectroscopic measurements, the spectral resolution is limited by the detected photon number as the spatial resolution [7]. Consequently, only a few bright localizations have been utilized for high-quality spectral analysis. Zhang et al. avoided this limitation by using an architecture with fully-connected layers to classify the spectra [41]. In their method, the full spectra obtained by SR-SMLM served as input for the network, and the network output was a color channel. To prepare the training data, single-molecule emission spectra of typical far-red dyes obtained by SR-SMLM were used. In the experiment, two spectrally similar dyes—AF647 and CF660—were chosen to validate their method, and their single-molecule spectra were assigned as the ground truth. By demonstrating two-color STORM images of AF647-labeled mitochondria and CF660-labeled microtubules, Zhang et al. showed that this method achieved a two-fold improvement in spectral data utilization as well as a ten-fold reduction in misclassification, compared to the previously used pixel-to-wavelength converted spectral mean method. This method is expected to lead to further multiplexed simultaneous SR-SMLM imaging when combined with optical implementation and fluorophore design [41].

## 5. Perspectives on Future Deep-Learning-Based SMLM Analysis

Deep learning has had a huge impact on computer vision and image processing and has become an emerging powerful tool for image analysis. Recently developed super-resolution microscopic techniques have also taken advantage of deep learning. Various effective and well-performing neural network architectures have been suggested for SMLM image data analysis based on the type of input data and target output data, and their outstanding performance has been continually reported over the past four years. First, by replacing the time-consuming existing MLE-based fitting algorithms, deep-learning-based algorithms can accelerate single-molecule localization and image reconstruction in SMLM. Furthermore, they enable the reconstruction of high-density super-resolution images from a relatively small number of imaging frames, thereby enhancing the time resolution of the SMLM technique. Localization precision can also be improved by deep learning algorithms, particularly for the localization of multi-emitter PSFs, which have been limited by existing fitting algorithms. Finally, a deep-learning-based approach can be employed to extract various hidden molecular information, such as the axial location and emission color information of single molecules, eventually improving localization and spectral precision.

Although recent SMLM imaging studies have already achieved remarkable results, there are still many suggestions to extend these approaches to the next level. First, a more modernized architecture can be applied to SMLM image analysis. For example, CNN-based feature networks have evolved enormously to outperform simple convolution architectures in both speed and accuracy. Transformer-based feature networks are gaining increasing attention in vision tasks. The adaptation of these newly developed architectures is expected to improve localization precision. Furthermore, the transfer learning approach may be helpful in obtaining more stable and robust results. Owing to the limitation of the variations in training data for the target task, it is becoming standard to use a large amount of image data to initialize the weight parameter of the neural network in most computer vision algorithms. As described previously, many deep learning approaches in SMLM significantly depend on simulated data, which may cause challenges in obtaining a correct output from the experimental data. Transfer learning avoids this limitation, resulting in a more robust algorithm. In addition, other types of computer vision algorithms and methods can be applied. For example, object and key point detection algorithms can be used to localize each emitter in an image. A generative model and GAN can contribute to the construction of a training dataset and self-supervised learning strategy. A series of imaging frames can also be fed into the neural network as video data instead of a single image level. For video data, sequential information may be helpful for detecting target objects and further improving accuracy. Finally, an open benchmark dataset will lead to the overall advancement of this research field. All research discussed in this article used private data to demonstrate their results, which likely limited comparability with future studies by other researchers. For a precise comparison between approaches with similar objectives, an open dataset would be beneficial. This is expected to not only attract more attention from researchers who have difficulty accessing super-resolution images but also accelerate the further development of algorithms. 

Overall, the deep learning approach is anticipated to have a significant impact on single-molecule localization microscopy. We expect that these developments can be extended further with more diverse methods and experiments adapted from the established methods of deep learning research in the computer vision field. 

## Figures and Tables

**Table 1:** Comparison of reported studies on the deep-learning-based single-molecule localization image analysis.

Type	Name	Architecture	Algorithm	Input	Output	Training Data	Reference
Acceleration of single-molecule localization	DeepSTORM	Decoder-Encoder	Image Reconstruction	Camera Images with multiple PSFs	SR image in 2D	-Simulated images of emitters and microtubules -Experimental images of microtubules	[31]
	smNET	ResNet-like CNN	Regression	Individual Image of PSFs	3D coordinates of PSFs, orientation, etc.	-Simulated images of emitters -Experimental images of mitochondria and bead	[39]
	--	CNN	Regression	Individual Image of PSFs	3D coordinates of PSFs	-Simulated and experimental images of beads	[33]
	DeepLOCO	CNN + FC with Residual Connection	Regression	Camera Images with PSFs	3D coordinates of PSFs	-Simulated and contest data	[32]
Constructing high-density super-resolution image	ANNA-PALM	U-Net, GAN	Image Generation	Widefield image, Image Sequences with multiple PSFs	Super-resolved 2D image	-Simulated image of microtubules -Experimental images of microtubules, nuclear pores and mitochondria.	[35]
	--	CNN	Image Reconstruction	Image of individual PSFs	Super-resolved 2D image	-Experimental images of microtubules, mitochondria, and peroxisome.	[36]
Improvement of localization precision	BGnet	U-Net	Image Reconstruction	Images of Individual PSFs	Background and intensity of PSFs	-Experimental images of microtubules	[37]
Localization of overlapping PSFs	DECODE	U-Nets	Image Reconstruction	Image Sequences with multiple PSFs	3D coordinates of PSFs, Intensity, Background, Uncertainty	-Contest data -Experimental images of microtubules, golgi, and nuclear pore complex.	[19]
Extracting additional spectral information from PSF	--	FC	Classification, Regression	Individual Image of PSFs	Axial Position, Color	-Simulated images of emitters -Experimental images of beads, microtubules and mitochondria.	[38]
	--	CNN, Encoder–Decoder	(1) Classification, (2) Image Reconstruction	(1) Image of individual PSFs (2) Camera Images with multiple PSFs	(1) Color channel (2) Color channel, 2D coordinates of PSFs	-Simulated images of emitters -Experimental images of Qdots, microtubules, and mitochondria.	[40]
	--	FC	Classification	Full Spectra	Color Channel	-Experimental images of microtubules and mitochondria.	[41]
	DeepSTORM3D	CNN with skipped connection	Image Reconstruction	(1) Simulated point sources (2) Camera Images with multiple PSFs	(1) PSFs (2) 3D coordinates of PSFs	-Simulated images of emitters -Experimental images of telomeres	[42]

## Abbreviations

SRM	super-resolution fluorescence microscopic techniques
SMLM	single-molecule localization microscopy
PSF	point spread function
STORM	stochastic optical reconstruction microscopy
PALM	photo-activated localization microscopy
FPALM	fluorescence photoactivation localization microscopy
STED	stimulated emission depletion
RESOLFT	reversible saturable optical fluorescence transition
SIM	structured-illumination microscopy
SSIM	saturated structured-illumination microscopy
EM	electron microscopy
ReLU	rectified linear units
MLP	multilayer perceptron
MNIST	Modified National Institute of Standards and Technology
CNN	convolutional neural network
VGG	Visual Geometry Group
RNN	recurrent neural network
LSTM	long short term memory
GRU	gated recurrent unit
GAN	generative adversarial networks
RCNN	region convolutional neural network
MLE	maximum likelihood estimation
SR	super-resolution
SLM	spatial light modulator
SR-SMLM	super-resolution single-molecule localization microscopy
